# Self-Paced (Asynchronous) BCI Control of a Wheelchair in Virtual Environments: A Case Study with a Tetraplegic

**DOI:** 10.1155/2007/79642

**Published:** 2007-09-10

**Authors:** Robert Leeb, Doron Friedman, Gernot R. Müller-Putz, Reinhold Scherer, Mel Slater, Gert Pfurtscheller

**Affiliations:** ^1^Laboratory of Brain-Computer Interfaces, Institute for Knowledge Discovery, Graz University of Technology, Krenngasse 37, 8010 Graz, Austria; ^2^Department of Computer Science, University College London, Gower Street, London WC1E 6BT, UK; ^3^Sammy Ofer School of Communications, The Interdisciplinary Center, P.O. Box 167, Herzliya 08010, Israel; ^4^Catalan Institute of Research and Advanced Studies (ICREA), Polytechnic University of Catalunya, 08010 Barcelona, Spain

## Abstract

The aim of the present study was to demonstrate for the first time that brain waves can be used by a tetraplegic to control movements of his wheelchair in virtual reality (VR). In this case study, the spinal cord injured (SCI) subject was able to generate bursts of beta oscillations in the electroencephalogram (EEG) by imagination of movements of his paralyzed feet. These beta oscillations were used for a self-paced (asynchronous) brain-computer interface (BCI) control based on a single bipolar EEG recording. The subject was placed inside a virtual street populated with avatars. The task was to “go” from avatar to avatar towards the end of the street, but to stop at each avatar and talk to them. In average, the participant was able to successfully perform this asynchronous experiment with a performance of 90%, single runs up to 100%.

## 1. INTRODUCTION

Virtual reality (VR) provides an excellent training and testing environment for rehearsal of scenarios or events that are otherwise too dangerous or costly—or even currently impossible in physical reality. The technological progress in the last decade has made VR systems attractive for various research fields and applications ranging from aviation and military applications, simulation and training programs (where real-life training is too expensive or difficult to monitor and control), psychotherapy, and medical surgery. In particular, the area of medical rehabilitation exploits the possibilities and advances made available by VR systems, specifically the rehabilitation of motor functions [1, 2] including stroke rehabilitation (upper and lower extremity training) [3], spatial and perceptual motor training, Parkinson's disease, orthopedic rehabilitation [4], balance training, and wheelchair mobility [5]. A major finding in this field is that people with disabilities can perform motor learning in VR, which can then be transferred to reality [6, 7]. In some cases it is even possible to generalize to other untrained tasks including improved efficiency of virtual training and learning [1, 2, 8]. It is important to note that VR is not a treatment by itself, and therefore it is impossible to study whether it is effective or not for rehabilitation. Although VR rehabilitation was undertaken in patients with acquired brain injury or damage with some success [9, 10], it is rather a new technological tool, which may be exploited to enhance motor retraining.

Virtual environments (VE) have already been used as a feedback media for brain-computer interface (BCI) experiments. BCI technology deals with the development of a direct communication channel between the human brain and machines which does not require any motor activity [11, 12]. This is possible through the real-time analysis of electrophysiological brain signals recorded by electroencephalogram (EEG) or electrocorticogram (ECoG). Other than the EEG and ECoG, brain signals can also be recorded invasively by implanted electrodes in the brain. Voluntary mental activity (e.g., a sequence of thoughts) modifies bioelectrical brain activity and consequently the EEG and ECoG. A BCI is able to detect such changes and generate operative control signals. Particularly for people suffering from severe physical disabilities or are in a “locked-in” state, a BCI offers a possible communication channel. Recently, the BCI has been used to control events within a VE, but most of the previously conducted VR-BCI research is based on two types of visually evoked responses; either the steady-state visual evoked potential (SSVEP) [13] or the event-related P300 potential [14]. These methods typically force the subjects to perform a visual task which might be unnatural (e.g., to gaze at a blinking object). In contrast, no visual stimuli are necessary if oscillatory EEG components, modulated by specific mental strategies (e.g., motor imagery), are used for the BCI [11]. With such a system participants are able to navigate through VEs by imagination of hand or foot movements [15, 16]. Thereby, the EEG is analyzed in predefined time intervals (cue-based or synchronous BCI) and the participants can decide between two states (either go right/left or forward/stop), but only whenever they are triggered by the system. The disadvantage of such a synchronous BCI and of a BCI based on evoked potentials is that an external stimulus from the system is always necessary and that the system always makes a decision (out of a predefined set of choices, e.g., movement imaginations). Up to now, most of the existing BCI systems are operated in this synchronized (or cue-based) paradigm, but this is not the natural way of human-machine interaction.

Transferring the BCI from laboratory conditions towards real-world applications needs the identification of brain patterns asynchronously without any timing constraints: the computer is no longer in control of timing and speed but the user. An asynchronous (self-paced) BCI is continuously analyzing the ongoing brain activity, however, not only the intentional-control (IC) states have to be detected (e.g., motor imagery) but also the in-between periods, whereas the user is in a non-control state (NC, formerly called idling state). In the later, the user may be idle, daydreaming, thinking about something else, or performing some other action, but is not trying to control the BCI. Asynchronous BCIs are much more complicate than synchronous ones, nevertheless, the community is more and more addressing these problems [17 21]. A big challenge in case of asynchronous BCIs is the validation. The performance is mostly measured in percentage of successful switching (true positive rate, TP) between IC and NC (or between the different IC states) and percentage of false or not intended switching (false positive rate, FP). For computing the correct TP/FP rates, it is necessary to access the subjects “real” intend and to compare it with the BCI output. Unfortunately, this information is not directly accessible. So either the system is telling the user to perform a switch or the user is reporting immediately if a switch occurred correctly or not. In the first scenario, analogical to a cue-based (synchronous) application, the system and not the user is in control of the timing [22]. In the second scenario, verbal comments or keystrokes could be used to verify the correctness of the BCI output, nevertheless the execution of such response tasks is modifying the EEG and thereby influencing the BCI output as well. A different approach is to give the user a task, which is only accomplishable by having control over NC and IC states and measuring only the task performance. Thereby, no concrete values for the TP and FP rates are computable, but the definition of the task involves that a high number of TP and a low number of FP is necessary. This procedure has been applied in this paper.

In this case study we want to demonstrate that it is possible for a tetraplegic subject to intentionally control his wheelchair within virtual reality by self-paced motor imagery using an EEG-based BCI. The participant is placed inside a virtual street populated with avatars and the task is to “move” from avatar to avatar towards the end of a street by imagination of movements of his paralyzed feet. The reason for the VR-setup is that the visual-rich virtual street with the avatars ensured that the experiment is diversified and engaging but contains enough distraction as it would be in a real street. The defined experiment has a simple goal with clear tasks, nevertheless, no instructions or cues from the BCI are necessary. A minimized setup of one bipolar EEG recording should be enough for this asynchronous control under real-world-like VR conditions.

## 2. METHODS

### 2.1.The tetraplegic subject

Here, we report on a 33-year-old male tetraplegic subject. After a traumatic injury of the spinal cord in 1998, he has a complete motor and sensory lesion below C5 and an incomplete lesion below C4. During an intensive training period of approximately 4 months, he has learned to control the cue-based Graz-BCI. The training was carried out with different types of motor imagery (MI; left- and right-hand motor imageries, idling, and foot movement imaginations) because of the insufficient accuracy in the beginning. The MI had to be performed within 4 seconds following the cue-stimulus. Finally, his cue-based performance during right-hand versus foot motor imagery was between 90% and 100% (details about this training are reported elsewhere [23]). Specifically, the midcentral-focused beta oscillations with a dominant frequency of approximately 17 Hz allowed a brain-switch like application of a neuro-prosthesis [24, 25]. Thereby, he had to focus on a foot movement imagination over a period of 1 second (dwell time) to activate a trigger and initiate a grasp sequence. After each trigger, a refractory period of 5 seconds guaranteed that no further grasp sequence could be initiated. The same brain rhythms have been used in this work for the self-paced control of the VE.

### 2.2. Data acquisition and signal processing

One single EEG channel was recorded bipolarly 2.5 cm anterior and posterior to the electrode position Cz (foot representation area) of the international 10/20 system [26]. The ground electrode was positioned on the forehead (position Fz). The EEG was amplified (sensitivity was set to 50μV and the power-line notch filter was activated), bandpass filtered between 0.5 and 30 Hz with a bipolar EEG amplifier (g.tec, Guger Technologies, Graz, Austria) recorded and online processed with a sampling frequency of 250 Hz [27]. The recording and processing was handled by rtsBCI [28], based on MATLAB 7.0.4 (MathWorks, Inc., Natick, USA) in combination with Simulink 6.2, Real-Time Workshop 6.2, and the open source package BIOSIG [29].

A single logarithmic band power (BP) feature was estimated from the ongoing EEG by digital band-pass filtering the recording (Butterworth IIR filter of order 5, between 15 and 19 Hz), squaring, averaging (moving average) the samples over the past second, and computing the logarithm from this time series. A simple threshold (TH) was used to distinguish between foot movement imagination (intentional control, (IC)) and rest (non-control state (NC)). Whenever the band power exceeded the threshold, a foot MI was detected (see Figures 3(a)–3(e)).

An “idling” recording (approximately 120 seconds) without any foot movement imagination was recorded before the experiment for the calculation of the TH. The BP was calculated and the mean $\bar{x}$ and the standard deviation SD were extracted. The TH was set to

(1) $$\text{TH}=\overset{¯}{x}+3\cdot \text{SD.}$$

Unlike previous asynchronous studies no, dwell time (minimum time over threshold before the action is triggered) or refractory period (minimum time between two successful actions) was used [22, 24].

### 2.3. The virtual environment

**Figure 1:**
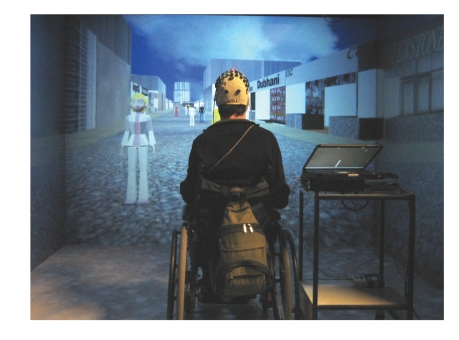
Picture of the virtual street populated with 15 avatars and the tetraplegic subject in his wheelchair in the middle of the multi-projection wall VR system. The subject was wearing the electrode cap with one bipolar channel connected to the BCI system (amplifier and laptop on the right side).

**Figure 2:**
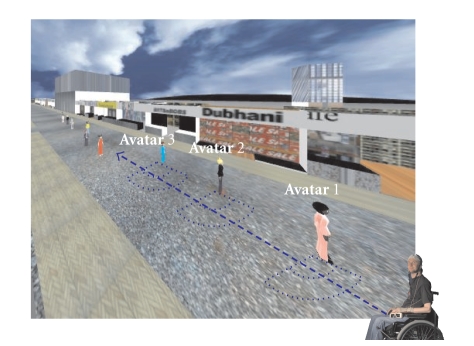
The task of the participant was to go from avatar to avatar towards the end of the street (outlined with a dashed line). The avatars were lined up and each avatar had its invisible communication sphere (drawn as dotted lines here). The subject had to stop within this sphere, not too close and not too far away from the avatar.

The participant was placed with his wheelchair in the middle of a multiprojection-based stereo and head-tracked VR system that commonly known as a “Cave” [30]. The particular VR system used was a ReaCTor (SEOS Ltd. West Sussex, UK) which surrounds the user with three back-projected active stereo screens (3 walls) and a front-projected screen on the floor (see Figure 1). Left- and right-eye images are alternately displayed at 45 Hz each, and synchronized with CrystalEye stereo glasses. A special feature of any multiwall VR system is that the images on the adjacent walls are seamlessly joined together, so that participants do not see the physical corners but the continuous virtual world that is projected with active stereo [31]. The application was implemented in DIVE [32] and the communication between the BCI and the VR occurred every 40 milliseconds via the Virtual Reality Peripheral Network (VRPN, [33]) communication protocol. The used VE was a virtual street with shops on both sides and populated with 15 virtual characters (avatars), which were lined up along the street (see Figure 2, [15]).

### 2.4. Experimental setup

**Figure 3:**
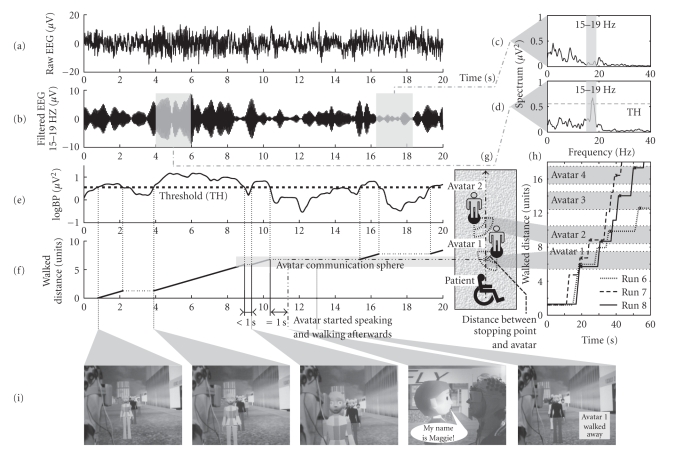
(a) Raw EEG during periods of foot MI and rest. (b) Bandpass-filtered (15–19 Hz) EEG. (c), (d) Power spectra of 2-second periods during rest (c) and foot MI (d). The frequency band and the threshold are indicated. (e) Logarithmic band power time course with threshold (TH). (f) Periods of moving and covered distance. The contact with avatar 1 occurred at second 11.4 after a 1 second pause within the communication sphere (because at second 8.9 the subject stopped shorter than 1-second). (g) Spatial placement of the avatars with corresponding communication sphere and direction of walking. The communication sphere of avatar 1—stopping range for the subject—is also marked with a gray rectangle (f). (h) Spatial-temporal tracking data of the first four avatars of three runs. The communication spheres of avatars are again indicated with a gray rectangle. The time and position of the contact with the avatar are marked with an “∗”. In run 7, the third avatar was missed. (i) Picture sequence before, during, and after the contact with avatar 1.

The task of the participant was to “move” from avatar to avatar towards the end of the virtual street (65 length units) by movement imagination of his paralyzed feet. Only during the time when the TH was exceeded (IC, foot MI detected), the subject moved forward (moving speed 1.25 units/second, see Figure 3(e)-3(f)). Every time he was short before passing an avatar, he had to stop very close to it. Each avatar was surrounded by an invisible communication sphere (0.5–2.5 units) and the subject had to stop within this sphere (see Figure 2 and Figure 3(g)). The size of the sphere was adequate to the distance for a conversation in the real world and corresponded to a stopping time slot of approximately 1.6 seconds. The avatar started talking to the subject, if he was standing still for one second within this sphere (see Figure 3(i)). After finishing a randomly chosen short statement (like “Hi,” “My name is Maggie,” “It was good to meet you,” etc.), the avatar walked away. Communication was only possible within the sphere; if the subject stopped too early or stopped too close to the avatar nothing happened. After a while, on his own free will, the subject could imagine another foot movement and started moving again towards the next avatar, till the end of the street was reached. The distance traversed depended only on the duration of the foot motor imagery, longer foot MI resulted in a larger distance than short bursts of MI. The 15 avatars were placed on the same positions in all ten experiments and the participant always started from the same point. The subject was encouraged to look around in the street during the experiment and to answer the statements of the avatars, like it would be in reality.

## 3. RESULTS

In two days, the tetraplegic subject performed ten runs and he was able to stop at 90% of the 150 avatars and talked to them. In four runs, he achieved a performance of 100% (see Table 1). In general, the distance between the avatar and the subject during talking was 1.81 ± 0.49 units, whereby, the communication range (allowed gap between avatar and subject) was 0.5 to 2.5 units. In the example given in Figure 3(f), the subject entered the communication sphere of avatar 1 (5.5–7.5 units) at second 8.3 and stopped at second 8.9 (6.1 units). Unfortunately, he started moving again at second 9.3, so the pause was below 1 second and, therefore, the stop did not activate the avatar. Nevertheless, he managed to stop again at second 10.4 (7.1 units), which was still in the communication sphere and at this time, he was standing still for longer than 1 second, so he correctly stopped at the avatar, which replied: “My name is Maggie” at second 11.4. At second 15.4, he started moving towards avatar 2. In general, it took him 6.66 seconds ± 4.85 to restart moving after the contact with the avatar.

In Figure 3(h)(h), spatial-temporal tracking data of the first four avatars of three runs are presented. In some runs, the subject started earlier with foot MI and walked straight to the avatar, whereby in other runs stops between the avatars occurred. Detailed information of all runs is given in Table 1. The duration of one run (each run lasted approximately 355 ± 60 seconds) depended only on the performance of the subject. In the Graz-BCI, the EEG is classified sample-by-sample and the EEG data revealed that foot motor imagery could be detected in 18.2% ± 6.4% of the run time. The averaged duration of MI periods was1.58 seconds ± 1.07, with a maximum of 5.24 seconds and a median of 1.44 seconds.

In 11 of the 15 missed avatars (of all runs), the subject stopped within the communication range, but the stopping time was too short (between 0.08 and 0.88 seconds, mean ±SD = 0.47 second ± 0.27 second). In Table 1, the number of these occurred stops is given in square brackets for each missed avatar. The stops occurred at 1.43 ± 0.47units before the avatar. At one avatar he stopped twice but both stops where too short (0.76 and 0.56 seconds).

### 3.1. Simulation with surrogate data: random walk simulation

For evaluation purposes, a “random walk” simulation was performed. The aim of this simulation was to demonstrate that only the intentional thought-based control of the subject allowed a successful completion of the task. No coincindentally created sequence should result in any correct accessed avatar. As surrogate data a random sequence has been used instead of the EEG for simulating IC (go) and NC (stop). The ratio of IC to NC was varied and always 10 000 repetitions were simulated. In the case with the same ratio as in the performed experiments (IC : NC = 0.182 : 0.818), no avatars were correctly accessed. It is clear that, if the surrogate data would contain only one go (IC) sample followed by, for example, thousands of stop (NC) samples and then the next go sample and so on, a completely perfect performance (contact with every avatar) would be produced, but the duration of the run would increase towards infinity. Therefore, several ratios were examined (0.5, 0.1, 0.05,… : 1) and almost all of them returned zero hits (correctly accessed avatars). The first avatar contact occurred at a ratio of 0.001 : 1 (IC to NC samples). For this test, the duration of the run had to be extended by 200 times (about 20 hours) and the number of correctly stopped avatars was 0.002 ± 0.015, compared to 13.5 ± 1.6 avatars within 355 seconds (about 6 minutes) in the performed experiment with the tetraplegic subject.

## 4. Discussion

The implementation of a self-paced (asynchronous) EEG-based BCI for the control of applications in virtual environment was successfully demonstrated with a tetraplegic subject. The patient was able to move forward in the virtual street and to stop at each avatar with a single bipolar EEG recording. The prominent induced centrally localized beta oscillations, allowed a very simple signal processing approach (compare Figure 3(c) with Figure 3(d)). In an interview, the subject confirmed that moving occurred only during periods of foot motor imagery, but he reported that it was hard to stop precisely. Specifically, when the avatars were placed more to the left or right, it was difficult to find the “correct distance” to the avatar. The instructions given to the participant before the experiment did not imply the restriction to perform the experiment as fast as possible, but to take his time to look around, talk to the avatars or enjoy the VR world. Therefore, no statement about the duration of the runs can be given.

In four runs, the subject was able to reach a performance of 100%, in all other runs, in minimum one avatar was missed. In most of the missed avatars (except two), the subject stopped either too shortly within the communication sphere or stopped close before (too early) or shortly after the sphere (too late). The reason for these missed avatars was the invisible communication sphere around the avatars, which was reported by the subject as the biggest disadvantage of this VE. So it was not clear for the subject where the sphere started or ended, especially when the avatars were placed further away from the middle of the street and the sphere was, therefore, very small. Sometimes he thought to be close enough, but maybe missed it by a hairbreadth, so an additional very small “step” (very short foot MI) was necessary to come inside the sphere. Unfortunately, it could happen that this step was too large (too long) and the sphere already passed by. Oscillatory EEG components need some time to appear and stop, so very short bursts (necessary for such small steps) are very unlikely to be produced. Maybe it would have been better to visualize the communication sphere or to change the appearance of the avatar (e.g., the color, the expression on the face, etc.) whenever the subject has entered the sphere. Nevertheless, the design of the experiment with necessary periods of IC (moving) and defined positions of NC (stopping close to the avatars) guaranteed that the performance during the asynchronous control could be verified. A drawback of the experimental design is that it forced the subject to be able in minimum to stop for 1 second (NC), but did not force the participant for shorter or longer periods of IC (no impact on the performance, just influencing the duration of a run). So good NC control was more crucial than IC control. Unfortunately, no values for TP and FP can be given for the experiment. Successful stops (90%) could be reported as TP and missed avatars (10%) as FN (false negative), but FP and TN (true negative) cannot be evaluated. The experiment itself required only periods of NC for stopping at the avatars and talking to them. Therefore, after reaching the last avatar, the BCI was not stopped and the subject was instructed to stand still and wait till the VE was shut down. In this period of NC no movement happened (duration between 8 and 93 seconds, mean = 44 seconds). The outcome of the simulation with surrogate data showed that only the intentional control of the subject allowed a successful accomplishment of the given task, almost all simulated data resulted in zero hits (no correct avatar contact).

The usage of a visually-rich VE with avatars, which were talking to the subject, ensured that the experiment was diverse and even distracting for the subject, somewhat like in the real world. Nevertheless, the subject was able to succeed with 90%. It is known that the development of skills or knowledge that are obtained while someone is in a VE can be transferred to real-world behavior and performance [6, 7]. Indeed, VEs have also been shown to reinforce the building of new neural pathways through imaginations or intention to move a paralyzed limb [1, 2]. For a person who is wheelchair-bound, VEs are especially attractive. First, simply using a VE that includes, for example, immersion in an almost all-surrounding stereo world [31] with the freedom to move at will can give such persons access to experiences that may be long forgotten (or which they have never had). Another advantage here is that the simulation power of VEs can be used to create virtual prototypes of new navigation or control methods, and give potential users experience of them in a safe environment, before they are ever built physically.

The next step would be to extend the BCI to more than one IC state, and thereby increase the degree of freedom and allow the subject to choose the direction of moving, for example, by imagining a left- or right-hand movement [34]. In the future, the final goal will be to control a real wheelchair in a real street. This could be supported by applying a similar procedure as Millán [35] reported during the control of a miniaturized robot through an 80 × 60cm representation of an empty flat. Thereby, the BCI was sending only high-level commands (forward, right, follow left wall, etc.) every 0.5 second to a finite state automation. The robot was executing the high-level command (e.g., turn right at next occasion) autonomously using its on-board sensors (infrared proximity sensors for obstacles detection) and was continuing the command till the next high-level command was sent. Although the difficulties and challenges are more on the side of the robot/wheelchair as on the side of the BCI, the feasibility of a successful completion of such real-world navigation task is increased.

## 5. Conclusion

For the first time, it was demonstrated that a tetraplegic subject, sitting in a wheelchair, could control his movements in a VE by the usage of a self-paced (asynchronous) BCI based on one single EEG recording. The usage of a visual-rich VE with talking avatars ensured that the experiment is diversified and engaging but contains enough distraction as it would be in a real street. Controlling a VE (e.g., the virtual wheelchair) is the closest possible scenario for controlling the real wheelchair in a real street, so virtual reality allows patients to perform movements in a safe environment. So a further step of transferring the BCI from laboratory conditions towards real-world applications could be performed.

## Figures and Tables

**Table 1:** Positions and times of the contacts between the subject and the avatars of all runs. The position is given in units and the time (in seconds) of the speaking avatar is given in brackets behind the position. In the first two columns, the number of the avatar (No.) and the spatial placement of the avatar (AvPos) are shown. In case of missed avatars, the number of occurred stops within the communication sphere is given in square brackets. In the last row, the performance (perf., in %) of each run is specified.

No.	AvPos	Run 1	Run 2	Run 3	Run 4	Run 5	Run 6	Run 7	Run 8	Run 9	Run 10
1	8	6.7 (48.2)	6.3 (56.7)	6.8 (33.0)	5.9 (12.0)	5.9 (14.8)	5.6 (19.5)	6.6 (20.0)	5.6 (19.8)	6.6 (53.4)	5.9 (18.8)
2	11	9.3 (65.3)	9.2 (76.0)	9.1 (43.7)	9.0 (20.8)	9.0 (22.1)	9.7 (36.6)	9.0 (25.4)	8.7 (31.7)	10.0 (60.7)	9.8 (52.0)
3	15	13.7 (84.6)	12.7 (98.5)	13.2 (55.9)	12.4 (31.6)	— [1]	12.7 (54.0)	— [1]	14.1 (42.4)	— [1]	13.1 (56.4)
4	18	15.7 (92.1)	16.6 (111.7)	17.2 (67.8)	16.6 (43.3)	15.4 (37.0)	16.4 (66.7)	16.4 (40.1)	17.2 (51.0)	15.6 (75.9)	16.1 (61.4)
5	22	20.5 (117.4)	20.3 (127.2)	20.0 (82.0)	20.6 (55.5)	19.5 (48.2)	— [1]	20.2 (45.4)	20.9 (61.2)	20.9 (99.0)	— [0]
6	32	30.2 (131.3)	30.6 (149.8)	29.7 (100.4)	29.9 (74.6)	— [1]	30.4 (96.6)	30.3 (59.3)	29.4 (83.0)	30.1 (113.3)	30.9 (78.6)
7	35	33.2 (150.3)	— [1]	33.6 (109.6)	33.5 (92.2)	— [1]	34.1 (113.0)	33.5 (72.2)	33.1 (101.9)	33.6 (122.5)	33.3 (84.4)
8	39	37.8 (175.0)	36.5 (180.6)	37.7 (120.8)	36.4 (97.7)	36.4 (85.9)	36.6 (119.5)	36.4 (80.3)	37.8 (117.2)	37.3 (135.0)	36.4 (90.6)
9	44	41.5 (182.0)	42.0 (199.6)	42.3 (136.6)	42.8 (113.3)	41.5 (96.3)	42.6 (150.5)	41.9 (92.8)	42.1 (126.8)	42.8 (142.6)	41.9 (98.2)
10	47	44.5 (198.9)	44.8 (215.7)	45.3 (146.6)	44.7 (122.8)	45.6 (112.3)	46.2 (175.8)	45.1 (101.4)	45.3 (139.3)	45.3 (150.6)	45.0 (108.3)
11	51	48.7 (223.5)	50.0 (244.5)	48.6 (159.6)	49.7 (134.8)	— [1]	—[0]	49.1 (110.3)	49.3 (153.1)	49.0 (162.7)	49.8 (131.5)
12	56	54.1 (251.7)	55.0 (272.4)	53.9 (172.7)	53.7 (143.7)	53.6 (147.9)	54.0 (206.7)	54.5 (127.4)	54.1 (167.8)	55.1 (182.4)	— [1]
13	60	57.9 (277.6)	58.0 (283.5)	58.4 (187.1)	58.0 (156.7)	57.5 (165.3)	— [2]	58.4 (135.7)	58.1 (181.1)	—[0]	58.2 (157.4)
14	63	62.2 (305.5)	61.8 (300.0)	61.1 (198.0)	60.5 (164.3)	61.3 (177.7)	61.6 (239.8)	60.5 (150.2)	61.0 (191.5)	—[0]	60.9 (163.8)
15	67	65.3 (355.8)	66.4 (322.2)	65.2 (213.7)	65.2 (175.8)	65.8 (198.7)	— [1]	64.9 (160.7)	65.1 (204.9)	64.9 (212.7)	64.6 (172.2)

Perf.	100%	93.3%	100%	100%	73.3%	73.3%	93.3%	100%	80%	86.6%
